# Cell-based immunotherapy for neurodegenerative disease: A promising avenue

**DOI:** 10.4103/NRR.NRR-D-25-00816

**Published:** 2025-12-30

**Authors:** Tomas J. Huerta, Valentina Urbina-Muñoz, Valentina Urra-Alvarez, Cristopher Villablanca, Luis S. Gomez-Perez, Barbara Saavedra, Tomás Contreras, René L. Vidal

**Affiliations:** 1 Center for Integrative Biology, Universidad Mayor, Santiago, Chile; 2 Center for Geroscience, Brain Health and Metabolism, Santiago, Chile; 3 Escuela Tecnología Médica, Facultad de Medicina y Ciencias de la Salud, Universidad Mayor, Santiago, Chile; 4 Escuela de Biotecnología, Universidad Mayor, Santiago, Chile; 5 Escuela Nutrición y Dietética, Facultad de Medicina y Ciencias de la Salud, Universidad Mayor, Santiago, Chile

**Keywords:** Alzheimer’s disease, amytrophic lateral sclerosis, cell therapy, Huntington’s disease, immunomodulator, immunotherapy, neurodegenerative diseases, Parkinson’s disease

## Abstract

Neurodegenerative diseases such as amyotrophic lateral sclerosis, Alzheimer’s disease, Parkinson’s disease, and Huntington’s disease are characterized by progressive neuronal loss and chronic neuroinflammation, with current treatments remaining largely symptomatic. This review explores the potential of cell-based immunotherapy as a disease-modifying strategy. Advances in stem cell biology and immune engineering have facilitated the development of therapies using mesenchymal stem cells, chimeric antigen receptor T cells, macrophages, regulatory T cells, modified macrophages, and monoclonal antibodies. These approaches aim to regulate immune mechanisms implicated in neurodegeneration, such as microglial activation, systemic inflammation, and immune checkpoint dysregulation. Notably, macrophage-mediated delivery systems, such as genetically modified cells expressing neurotrophic factors or antioxidant enzymes, have demonstrated neuroprotective effects. Likewise, emerging data support T-cell modulation and monoclonal antibody development as therapeutic targets in amyotrophic lateral sclerosis, Alzheimer’s disease, Parkinson’s disease, and Huntington’s disease. We highlight current preclinical findings, underlying mechanisms, and translational challenges, emphasizing that immunomodulatory cell therapies represent a promising avenue for precision medicine in neurodegenerative diseases.

## Introduction

Neurodegenerative diseases such as amyotrophic lateral sclerosis (ALS), Alzheimer’s disease (AD), Parkinson’s disease (PD), and Huntington’s disease (HD) represent a major global health challenge, with prevalence rising as the population age. These disorders are characterized by progressive neuronal loss, accompanied by chronic neuroinflammation, and immune response dysregulation, leading to irreversible functional decline. Despite decades of research, current treatments remain largely symptomatic, failing to halt or reverse disease progression.

Recent advances in immunology, immune engineering, and stem cell research have opened new avenues for therapeutic intervention, particularly through cell-based immunotherapy. This approach aims to modulate mechanisms implicated in neurodegeneration, including microglial activation, systemic inflammation, and immune dysregulation. The development of therapies using mesenchymal stem cells, chimeric antigen receptor (CAR) T cells, regulatory T cells, modified macrophages, and monoclonal antibodies has shown promise in preclinical models, offering potential strategies for neuroprotection, symptomatic relief, and modified disease progression. However, translating preclinical success into effective clinical therapies remains a significant challenge.

In this review, we examine current preclinical findings, underlying molecular mechanisms, and translational obstacles in the field of cell-based immunotherapy. By integrating recent discoveries, we highlight the potential of precision medicine for neurodegenerative diseases, emphasizing the need for continued research to redefine therapeutic strategies and improve patient outcomes.

## Search Strategy

For this narrative review, all references were retrieved using bibliographic databases such as PubMed (https://pubmed.ncbi.nlm.nih.gov) and Google Scholar (https://scholar.google.com). Articles published within the last 25 years were considered, with particular emphasis on the past 5 years. The primary keywords included: cell-based therapy, cell-based immunotherapy, immunotherapy, macrophage-based immunotherapy, lymphocyte-based immunotherapy, neurodegenerative disease, amyotrophic lateral sclerosis, Alzheimer’s disease, Parkinson’s disease, and Huntington’s disease.

## Cell-Based Therapy as a Disease-Modifying Therapy

Cell therapy refers to the administration of functional cells to promote tissue regeneration or modulate pathological processes. It is classified as autologous or allogeneic, depending on whether the donor and recipient are the same or different individuals (Gage, 1998; Mount et al., 2015; Cossu et al., 2018). Moreover, cell therapies are categorized according to their potential to transform into different cell types. Pluripotent cells can differentiate into any cell type in the organism, while multipotent cells are restricted to lineage-specific, as they can only be transformed into a cell from the same lineage (Pirsadeghi et al., 2024; Boopathy et al., 2025).

In recent decades, stem cells have emerged as the primary cell type used in these therapies due to their capacity for self-renewal and ability to differentiate into multiple cell lineages (Jovic et al., 2022). These cells can be collected from several tissues, such as skin, cornea, and adipose tissue, through biopsy, or isolated from blood samples (Jovic et al., 2022; Na et al., 2023). Once obtained, most of these cells require *ex vivo* culture and expansion to reach a therapeutically effective quantity (Green, 1991; Pellegrini et al., 2013; Ricardo et al., 2013; Domenis et al., 2015).

Similarly, embryonic stem cells and induced pluripotent stem cells hold significant therapeutic potential. Established protocols allow for the differentiation of both embryonic stem cells and induced pluripotent stem cells into various neural cell types, using both monolayer and organoid cultures (Takebe et al., 2013; Shi et al., 2017; Engle et al., 2018; Nicholson et al., 2022), a process that remains one of the most challenging and advanced aspects of developing this technology.

Mesenchymal stem cells are also widely used in cell therapy research. They offer several advantages, including efficient *in vitro* growth and the potential for autologous transplantation (Li et al., 2023). Mesenchymal stem cells exhibit strong neurotrophic properties and support various regenerative mechanisms, particularly in neurovascular contexts. These mechanisms include angiogenesis, anti-inflammatory and anti-apoptotic effects, as well as the promotion of neurogenesis and cell differentiation (Wu et al., 2022; Li et al., 2023). Mesenchymal stem cells are also isolated from adipose tissue, peripheral blood, and the umbilical cord. The latter is considered an ideal source, offering cells with a broad differentiation capacity, ease of access, and suitability for autologous use. However, umbilical cord blood cells tend to have a short lifespan, with only a small fraction persisting in recipient tissues post-transplant (Liu and Cheung, 2020).

Recently, the focus of cell therapy approach has shifted to immune cells (here referred to as immune cell therapy), which naturally travel through the body and target specific sites (Anderson et al., 2021; Granhoj et al., 2022; Labanieh and Mackall, 2023; Gao et al., 2024; Han et al., 2024). One example is CAR cell therapy, which involves genetically modifying a patient’s immune cells with engineered receptors composed of an extracellular domain derived from monoclonal antibodies and intracellular signaling domains that activate immune responses upon antigen recognition (Labanieh and Mackall, 2023; Sun et al., 2024). Conversely, B cells play a central role in autoimmune diseases through both antibody-dependent and antibody-independent mechanisms, such as producing autoantibodies, presenting antigens to T cells, and secreting proinflammatory cytokines (Abeles et al., 2024). Cell therapies targeting B cells, especially monoclonal antibodies such as anti-CD20 (rituximab) (Morschhauser et al., 2018; Yang and Liu, 2021; Mankikian et al., 2023) and anti-CD19 (Inebilizumab) (Cree et al., 2019; Rensel et al., 2022; Stone et al., 2025), have shown significant success, though challenges remain in eliminating long-lived plasma cells. Macrophages are another immune cell type being studied due to their rapid response to environmental signals and high plasticity, playing a key role in numerous human diseases (Tian et al., 2022; Na et al., 2023; Li et al., 2024).

As previously noted, cell therapy utilizes diverse cell sources and types and has been applied across multiple medical fields, including autoimmune diseases, cancer, and neurodegenerative disorders, among others. In this review, we highlight the potential of immune cell therapy in treating the most prevalent neurodegenerative diseases.

## Immune Cell Therapy as a New Therapeutic Approach

Immune cell therapy represents a transformative strategy for cancer treatment and other immune-mediated disorders, including neurodegenerative diseases, harnessing the innate and adaptive capabilities of the immune system to recognize and eradicate pathogenic organisms and aberrant cells, including tumor and autoreactive immune cells (Demaria et al., 2019; Yi et al., 2022; Parikh and Lonial, 2023; Yan et al., 2023). Unlike conventional therapies, which often lack specificity and are associated with systemic toxicity, immune cell-based strategies offer precision, adaptability, and durable immune memory. The past decade has observed remarkable clinical success, particularly with the advent of CAR T cell therapy for hematological malignancies (Haslauer et al., 2021; Sterner and Sterner, 2021; Pasqui et al., 2022; Zhang et al., 2022b). Furthermore, ongoing advancements in gene editing, cell engineering, and biomaterials have expanded the therapeutic potential of natural killer cells (Wu et al., 2020; Vivier et al., 2024), dendritic cells (DCs) (Gardner and Ruffell, 2016; Peng et al., 2021), tumor-infiltrating lymphocytes, and stem cell–derived immune effector cells (Demaria et al., 2019).

Immunotherapy is currently based on reprogramming the immune system to target complex diseases through a range of strategic interventions. The first involves direct immunomodulators, a broad category that includes cytokines, interleukins, and other signaling molecules responsible for fine-tuning immune responses, either enhancing or attenuating activity depending on the physiological context (Leonard and Lin, 2023; Guo et al., 2024). Second are monoclonal antibodies, highly specific agents that label target cells for immune-mediated destruction (Tsao et al., 2021; Manso et al., 2023). A third key approach is the use of immune checkpoint inhibitors, which work by releasing the inhibitory signals on T lymphocytes, thereby restoring their ability to recognize and attack tumor cells that have developed mechanisms to evade immune surveillance (Shiravand et al., 2022). Additionally, oncolytic viruses are another innovative strategy, which selectively infect and lyse abnormal cells from within, while simultaneously releasing cellular signals that trigger the recruitment and activation of immune cells (Kaufman et al., 2015; Tsao et al., 2021; Wang et al., 2023). Unlike conventional vaccines, therapeutic vaccines are designed not to prevent disease, but to train the immune system to recognize and eliminate internal threats, such as tumor-specific antigens or toxic proteins (Sulzer et al., 2017; Saxena et al., 2021; Pesch et al., 2024; Gonzalez-Artero et al., 2025). Finally, adoptive cell therapy represents a leading approach in personalized immunotherapy, involving the extraction, genetic modification, and reinfusion of autologous immune cells to enhance their activity against pathological targets (Rosenberg and Restifo, 2015; Rampotas and Roddie, 2025).

Although initially emerged as a revolutionary strategy in cancer treatment, immunotherapy is no longer confined to oncology. Advances in immune modulation have broadened its application to other complex diseases, including autoimmune disorders, chronic infections, and even neurodegenerative conditions. For instance, recent studies have explored the use of CAR-T cell therapies in autoimmune diseases and infectious pathologies with promising results (Labanieh and Mackall, 2023; Gao et al., 2024). Additionally, immune checkpoint blockade (originally designed for cancer) has shown therapeutic effects in preclinical models of AD, suggesting potential benefits in neurodegeneration (Saetzler et al., 2023; Gao et al., 2024).

In the next section, we will summarize the evidence supporting the development of cell-based immunotherapy strategies for neurodegenerative diseases, focusing on ALS, AD, PD, and HD.

## Immune Cell Therapy for Neurodegenerative Diseases

Neurodegenerative diseases such as ALS, AD, PD, and HD are characterized by progressive neuronal loss and chronic neuroinflammation. Traditional therapeutic strategies have focused on symptomatic management or targeting protein aggregates such as amyloid-β or α-synuclein (α-syn). However, growing evidence implicates the immune system, particularly innate immune cells such as microglia and adaptive immune components in disease progression, making immunomodulatory cell therapy a promising frontier in the development of disease-modifying treatments (Lv et al., 2024; Zhao et al., 2025).

In the central nervous system (CNS), microglia are the resident innate immune cells and play a key role in the regulation of homeostasis in the brain (Liddelow et al., 2017; Lv et al., 2024; Zhao et al., 2025). Under pathological conditions, microglia adopt a pro-inflammatory phenotype that exacerbates neuronal injury via the release of cytokines such as interleukin-1β (IL-1β), IL-6, and tumor necrosis factor-alpha (TNF-α) (Lv et al., 2024; Zhao et al., 2025). This transition is often driven by damage-associated molecular patterns, misfolded proteins, and altered neuronal signals. Disease-associated microglia, a distinct subtype linked to neurodegeneration, have been shown to accumulate around amyloid plaques in AD and contribute to both neuroprotection and pathology depending on disease stage and context (Heneka et al., 2015; Liddelow et al., 2017; Lv et al., 2024).

Systemically, aging promotes “inflammaging,” a state of chronic low-grade inflammation marked by elevated circulating inflammatory markers. These systemic cytokines can cross a compromised blood-brain barrier and further activate glial cells, generating a feedback loop of sustained inflammation (Cicolari et al., 2021; Jurcau et al., 2024; Rim et al., 2024). Additionally, dysregulation of the complement cascade, especially complement C3, has been associated with AD progression, highlighting the crosstalk between peripheral and CNS immunity (Wu et al., 2019; Wei et al., 2021; Zhou et al., 2023). The adaptive immune system also plays a role in neurodegeneration. T cells infiltrate the CNS in neurodegenerative conditions, and their phenotypes (e.g., pro-inflammatory *versus* regulatory) significantly influence disease outcomes. For instance, regulatory T cells (Tregs) have shown neuroprotective effects in models of PD and AD by suppressing excessive immune activation, while effector T cells may exacerbate neuronal damage through cytokine release and microglial recruitment (DeMaio et al., 2022; Park et al., 2023; Yeapuri et al., 2023).

Importantly, genetic studies have identified numerous immune-related risk genes for neurodegenerative diseases, including TREM2, CD33, HLA variants, and components of the NLRP3 inflammasome, reinforcing the causal role of immune pathways in disease etiology (Ulland et al., 2017; Witoelar et al., 2017; Li et al., 2021; Siokas et al., 2021; Yi et al., 2023; Rachmian et al., 2024).

Taken together, neurodegenerative diseases share several pathological features, including immune dysregulation, a key driver of neurodegeneration shaped by dynamic interactions between central and peripheral immune systems, genetic susceptibility, and environmental factors. Additionally, genetic diversity and region-specific vulnerability add layers of complexity, particularly when designing effective immunotherapeutic strategies. Although it remains in early clinical translation, evidence from preclinical models and initial trials supports further investigation. In ALS, both peripheral macrophages and lymphocytes have been identified as contributors to pathogenesis. Notably, lymphocyte-based approaches have shown promising results in preclinical and clinical studies. In contrast, in AD, strategies targeting peripheral macrophages and lymphocytes aim to eliminate β-amyloid oligomers and phosphorylated tau; however, few have entered clinical trials, and outcomes remain controversial. In PD, therapies primarily focus on modified macrophages and lymphocytes to alleviate oxidative stress, neuroinflammation and reduce α-syn aggregation in preclinical models, yet none have progressed to clinical stages. In HD, a limited number of immunotherapeutic approaches have been proposed to restore immune cell function by depleting mutant huntingtin (mHtt) or employing anti-mHtt intrabodies, but none have been tested in patients. Advancing our understanding of CNS immunobiology is essential for realizing the full potential of these therapies. Unraveling the complex neuro-immunological landscape is critical to developing targeted immunomodulatory strategies that modify disease progression rather than merely alleviate symptoms.

The following sections will highlight current cell-based immunotherapy approaches for prevalent neurodegenerative diseases, including ALS, AD, PD, and HD. We will examine their mechanisms of action, preclinical and clinical efficacy, limitations, and future perspectives.

### Immune cell therapy on amyotrophic lateral sclerosis

ALS is a progressive, incurable neurodegenerative disorder marked by the gradual loss of motor neurons in the motor cortex, brainstem, and spinal cord, resulting in muscle function decline and impairments in mobility, speech, swallowing, and respiration (Dugger and Dickson, 2017; Feldman et al., 2022). Life expectancy following ALS diagnosis averages 2 to 5 years, with respiratory failure being the leading cause of death. The estimated incidence is two new cases per 100,000 individuals annually, with onset typically occurring between ages 55 and 65 (Talbott et al., 2016).

ALS is a highly complex pathology with two recognized forms: hereditary and sporadic. Approximately 10% of cases are hereditary (also referred to as familial or genetic). Among familial cases, 25% to 40%, and a small percentage of sporadic cases, are linked to mutations in the *C9orf72* gene, which encodes a protein expressed in motor neurons and brain nerve cells (Talbott et al., 2016). Additionally, 12% to 20% of familial cases are associated with mutations in the superoxide dismutase 1 (*SOD1*) gene, which encodes the enzyme copper-zinc superoxide dismutase 1 (Dugger and Dickson, 2017; Feldman et al., 2022).

Moreover, in ALS patients and preclinical models increased levels of pro-inflammatory cytokines/proteins (e.g., monocyte chemoattractant protein-1, IL-8, and C-reactive protein [CRP]) in serum and cerebrospinal fluid (CSF) have been detected (Fiala et al., 2010; Ryberg et al., 2010; Beers et al., 2011a; Kawaguchi-Niida et al., 2013), therefore, recent research has focused on both innate and adaptive immune responses in ALS with particular emphasis on the roles of microglia, macrophages, T and B lymphocytes, and the impact of cytokine release and autoantibodies in disease progression (Puentes et al., 2016; Beers and Appel, 2019; Mimic et al., 2023; Calma et al., 2024). Modulating these immune responses presents a promising avenue for developing unconventional therapeutic strategies against ALS (Calma et al., 2024).

#### Macrophage-based therapy in amyotrophic lateral sclerosis

A contemporary perspective suggests that ALS involves a non-cell-autonomous component in which glial cells contribute to motor neuron degeneration (Dugger and Dickson, 2017; Feldman et al., 2022). Notably, manipulating or modifying the microglia population, the CNS macrophages, affect disease progression and survival, as observed in ALS preclinical models (Boillee et al., 2006; Beers et al., 2008; Martinez-Muriana et al., 2016). However, in these studies, the entire myeloid lineage, including peripheral monocytes and macrophages, was manipulated, yet the observed effects were attributed solely to microglia. Thus, the individual contributions of peripheral monocytes/macrophages remain uncertain. Evidence strongly suggests that circulating monocytes/macrophages are deregulated in ALS. In preclinical models, these cells become activated prior to disease onset, with their recruitment to the spinal cord, but not the brain, correlating within neuronal loss (Chiu et al., 2009; Butovsky et al., 2012; Shiraishi et al., 2021). Similarly, in ALS patients, alterations in monocyte subtypes have been observed, including an increase in CD14^++^CD16^−^ monocytes and a decrease in CD14^+^CD16^++^ monocytes, compared to healthy individuals (Butovsky et al., 2012; Zondler et al., 2016). Monocytes derived from ALS patients exhibit impaired function, including decelerated phagocytosis and altered adhesion-migration behavior. Additionally, gene expression analysis showed dysregulation in several inflammatory-associated genes, including *CCR2*, *HNRNPA1*, *IL-8*, and *CCR1* (Zhang et al., 2006; Zondler et al., 2016). Some of these genes have been implicated in ALS pathology (Zondler et al., 2016) and proposed as early biomarkers (Ryberg et al., 2010; Lehnert et al., 2014; Butovsky et al., 2015).

**Figure 1:**
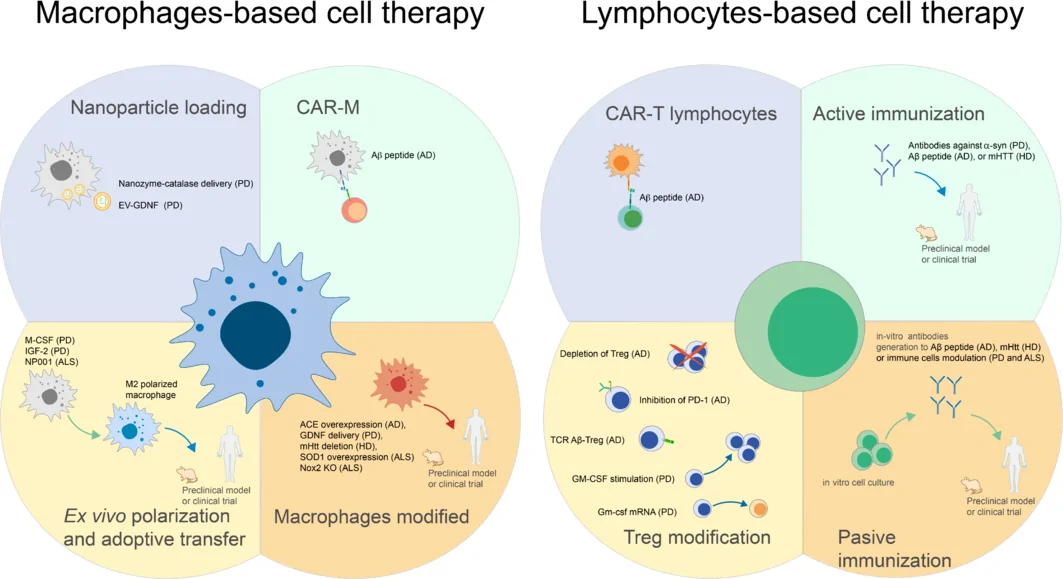
Current cell-based immunotherapies in neurodegenerative disease. A schematic summary of existing cell-based immunotherapies for neurodegenerative disease, Macrophage-based strategies include nanoparticle loading (developed for PD), CAR-macrophages (developed for AD), *ex vivo* polarization and adoptive transfer (developed for PD and ALS), and modified macrophages and adoptive transfer (developed for AD, PD, HD, and ALS). Lymphocyte-based strategies include chimeric antigen receptor lymphocytes (CAR-T) (Developed for AD), active and passive immunization (developed for AD, PD, HD, and ALS), and Treg modification (developed for AD, PD, and ALS). These approaches leverage immune system cells for therapeutic intervention in neurodegeneration. α-syn: α-Synuclein; ACE: angiotensin-converting enzyme; AD: Alzheimer’s disease; ALS: amyotrophic lateral sclerosis; Aβ: amyloid-β; CAR: chimeric antigen receptor; CAR-M: CAR-macrophages; EV: extracellular vehicle; GDNF: glial cell line–derived neurotrophic factor; HD: Huntington’s disease; IGF-2: insulin-like growth factor 2; KO: knockout; M-CSF: macrophage colony-stimulating factor; mHtt: mutant huntingtin; PD: Parkinson’s disease; SOD1: superoxide dismutase 1; TCR: T-cell receptor; Treg: regulatory T cells.

A key debate in ALS concerns the potential of bone marrow (BM)–derived monocytes/macrophages to infiltrate the CNS during disease, presenting a possible disease-modifying therapeutic strategy. A pilot trial using allogeneic hematopoietic stem cell transplantation in patients with sporadic ALS found no evident clinical benefits. However, it was suggested that donor-derived hematopoietic cells could enter the CNS, primarily at the site of motoneuron pathology, and engraft as immunomodulatory cells. These findings propose that monocyte/macrophages delivery to these areas could offer therapeutic potential (Appel et al., 2008). Furthermore, peripheral monocyte/macrophages infiltration into the spinal cord has been observed in ALS patients and preclinical models, and correlates with motor neuron survival (Graves et al., 2004; Zondler et al., 2016), as this is associated with recruitment of anti-inflammatory monocytes/macrophages (Shiraishi et al., 2021). Following this concept, BM transplantation (30 × 10^6^ cells; intraperitoneally injection) resulted in minimal infiltration of peripheral immune cells into the CNS of ALS preclinical mice, potentially impacting lifespan and neuronal survival (Corti et al., 2004; Solomon et al., 2006). Additionally, after-transplantation, these cells primarily populated the CNS microvasculature, whereas spinal cord microgliosis in mutant SOD1 mice, a preclinical model of ALS, was driven by resident microglia expansion rather than recruitment of BM-derived (Lewis et al., 2009). However, this phenomenon was later attributed to irradiation, as parabiosis, an alternative to irradiation/BM grafting, revealed no infiltration of peripheral monocytes into the spinal cord of ALS mice (Ajami et al., 2007; Mildner et al., 2007; Lewis et al., 2009). Nonetheless, Chiot et al. (2020) demonstrated that macrophages along peripheral motor neuron axons in ALS mouse models and patients respond to neurodegeneration. Moreover, in the ALS (mutant (m)SOD1^G93A^) mouse model, peripheral myeloid cell infiltration into the spinal cord was limited and dependent on disease duration. Interestingly, replacing wild-type (WT)- or mSOD1^G93A^- derived peripheral macrophages (5 × 10^6^ cells; intravenous injection) did not yield differences in nerve macrophages activation, microglial response, motor neuron degeneration, or T lymphocyte infiltration in mSOD1^G93A^ mice or WT. However, modified macrophages with overexpression of SOD1 or NADPH oxidase 2 (Nox2) knockdown reduced both peripheral macrophage and microglial activation, delayed symptom onset, and improved neuronal survival (Chiot et al., 2020; **Figure 1**). The survival-promoting effect of grafting modified macrophages appears linked to a shift in inflammatory profile of macrophages and an inversion of microglial responses toward neuronal support (Chiot et al., 2020). Despite ongoing debate, the pivotal role of peripheral monocytes/macrophages in ALS pathology is evident, and the therapeutic potential warrants further investigation to determine the most effective strategy.

Another approach using modified macrophages involves immunomodulators that selectively target these immune cells. NP001 is a chlorite-based, non-toxic intravenous compound and is considered a potential immunotherapy for ALS, designed to restore balance in a dysfunctional innate immune system (Jiang et al., 2022; Zhang et al., 2022a; Forrest et al., 2024; **Figure 1**). NP001 acts as a prodrug, converting to taurine chloramine upon contact with heme-associated iron, thereby inhibiting the nuclear factor kappa B (NF-κB) signaling pathway and inducing an anti-inflammatory profile, phagocytic, and wound-healing phenotype in macrophages (Giese et al., 2004). Although a phase 2a trial suggested NP001 could slow disease progression in patients with elevated CRP levels and demonstrated a favorable safety profile, the subsequent phase 2b trial did not meet its primary efficacy endpoint (Miller et al., 2015b). After 6 months, no statistically significant differences in ALS Functional Rating Scale-Revised (ALSFRS-R) scores or vital capacity were observed between the placebo and NP001 groups in a cohort of 138 American and Canadian patients with elevated plasma CRP levels (Miller et al., 2022). However, *post-hoc* analysis revealed that NP001 significantly slowed, or even halted, disease progression in a specific patient subgroup. Among patients aged 40–65 years, with disease onset within the previous three years and CRP levels above 1.13 mg/L, ALSFRS-R decline was reduced by 36%, while vital capacity loss was slowed by 51% (Miller et al., 2022). Notably, analysis of two NP001 phase 2 clinical trials suggests that NP001 is more effective in ALS patients with a body mass index ≥ 25, representing 70% of trial participants, and age ≤ 65 years at baseline. This subgroup showed significantly slower vital capacity decline and extended overall survival (Zhang et al., 2025). Recent findings indicate that NP001 slows ALS progression by regulating microbial translocation, as plasma biomarkers associated with microbial activity, including lipopolysaccharide, lipopolysaccharide-binding protein, IL-18, hepatocyte growth factor, and soluble CD163, were significantly reduced in NP001-treated patients compared to controls (Zhang et al., 2022a), proposing a systemic effect of the therapy. The evidence highlights NP001 as a promising treatment for ALS, though further studies are warranted.

While macrophage-based therapies have demonstrated efficacy in preclinical models, and some are now being tested in clinical trials, identifying appropriate patient candidates and their immunophenotype remains crucial for effective treatment and requires further investigation.

#### Lymphocyte-based therapy in amyotrophic lateral sclerosis

ALS patients exhibit immune system dysfunction, including impairment of the adaptive immune response. Immunoglobulin (Ig) G (IgG) and IgM deposits are found in motor neurons of the lumbar cord in postmortem samples and in blood from ALS patients and preclinical models (Chiu et al., 2009). Additionally, CD4^+^ and CD8^+^ T cells display altered function and phenotype, with CNS infiltration occurring at late disease stages (Chiu et al., 2008; Alsuliman et al., 2016). Patients with sporadic ALS show adaptive immune alterations in blood, including elevated CD4^+^ cell levels and reduced CD8^+^ T lymphocytes, attributed to decreased expression of the anti-apoptotic molecule Bcl-2. In mSOD1^G93A^ mice lacking functional T cells or CD4^+^ T cells, accelerated motor neuron degeneration is observed, accompanied by attenuated morphological markers of gliosis, increased mRNA levels for proinflammatory cytokines such as IL-6 and NOX2, and decreased levels of trophic factors, such as insulin-like growth factor 1 (IGF1) and glial glutamate transporters. Also, BM-transplants reconstituted mice with T cells, prolonged survival, suppressed cytotoxicity, and restored glial activation (Beers et al., 2008, 2011b). Moreover, endogenous T lymphocytes, primarily Tregs lymphocytes (CD4^+^CD25^+^ and CD25^+^FoxP3^+^), increase during the early stages of the disease, enhancing IL-4 and IL-10 expression and promoting an anti-inflammatory microglia phenotype. As the disease progresses, this lymphocyte population declines, possibly due to the loss of FoxP3 expression (Beers et al., 2011b). As proof of concept, the passive transfer (2 × 10^7^ wild-type or mSOD1 CD4^+^ T lymphocyte; intravenous injection) of inactive WT CD4^+^ T cells into an ALS preclinical mouse model lacking functional T cells (mSOD1/RAG2^−/−^) prolonged survival and lengthened disease duration. Furthermore, adoptive transfer of polyclonal-activated WT CD4^+^CD25^+^ Treg or CD4^+^CD25^−^ T effector (1 × 10^6^ cells; intravenous injection every 6 weeks at 7, 13, and 19 weeks of age) extended longevity and mitigated motor deficits. Interestingly, Tregs delayed clinical symptom onset, whereas effector T cells prolonged the latency from onset to late-stage disease (Banerjee et al., 2008), suggesting that a combined administration of both cell types may be more effective in treating ALS (**Figure 1**).

IL-2 modulates Treg proliferation and function by upregulating primarily CD25 and FoxP3 (Alsuliman et al., 2016; Ye et al., 2018). Several clinical trials using a combination strategy of autologous infusions of expanded Tregs combined with subcutaneous low-dose IL-2 were carried out (Thonhoff et al., 2024; **Figure 1**). The infusion of Treg/IL-2 was safe and well tolerated, and increased Treg numbers and suppressive function were higher in the active group of the randomized control trial after each infusion. Treg/IL-2 infusions slowed progression rates during early and later stages of disease, increasing Treg suppressive function correlated with slowing of disease progression per the Appel ALS scale for each patient (Thonhoff et al., 2018, 2022). This treatment demonstrated biological activity in individuals with ALS over 1 year, increasing both Treg suppressive function and cell number compared to baseline, and improving ALSFRS-R scores of patients. However, some concerns arise for the individuals who develop an increase of peripheral levels of inflammation and oxidative stress, which requires further investigation. A pilot clinical trial evaluated combination therapy with CTLA4-Ig, a fusion protein that modulates T-cell responses by upregulating expression of IL-2 and anti-apoptotic molecules (Ruderman and Pope, 2005). The treatment was safe and well-tolerated with no adverse events reported. During the first 24 weeks, the average rate of change in the ALSFRS-R remained unchanged; however, by week 48, individuals showed improvement. Treg suppressive function and numbers increased throughout treatment, while oxidized-LDL, 4-hydroxynonenal, IL-18, and neurofilament light chain levels decreased, suggesting an anti-inflammatory and antioxidant response (Thonhoff et al., 2024). Furthermore, a phase 2a clinical trial investigated the use of Aldesleukin (a recombinant form of IL-2) to expand Tregs in ALS patients. Aldesleukin was well tolerated in ALS patients receiving a 5-day course of once-daily subcutaneous injections. The treatment effectively increased both the number and frequency of Tregs. Furthermore, CD25 expression was upregulated on Tregs, potentially enhancing their sensitivity to both exogenous and endogenous IL-2 and improving therapeutic efficacy (Camu et al., 2020).

Another interesting approach using lymphocyte-based therapy is the development of antibodies that aim to regulate another immune system (**Figure 1**). Treatment with anti-Ly6C monoclonal antibody modulated the Ly6C^hi^ monocyte cytokine profile, reduced monocyte recruitment to the spinal cord, diminished neuronal loss, and extended survival (Butovsky et al., 2012). These findings where further corroborated in ALS patients, as the analogous monocytes (CD14^+^CD16^–^) exhibited an ALS-specific microRNA inflammatory signature like that observed in the ALS mouse model, linking the animal model and the human disease (Butovsky et al., 2012). Moreover, *in vitro* treatment with human IgG or CD95–Fc fusion proteins is able to rescue the altered monocyte subtype composition observed in ALS patients and an *in vivo* treatment with these antibodies also increase infiltration of monocytes and delay disease onset in a dose dependent manner (Zondler et al., 2016).

Integrin α5 has been proposed as a potential therapeutic target in ALS. This protein is expressed on immune cells in the brain (microglia) and on macrophages and natural killer lymphocytes in the spinal cord of ALS patients and preclinical models with amyotrophic lateral sclerosis. Monoclonal antibodies targeting this protein (5 mg/kg, intraperitoneal injection) in mSOD1^G93A^ mice, lead to a significant decrease in Iba1 immunoreactivity in microglia, improving motor symptoms and survival compared to those treated with an isotype control (Jiang et al., 2022; Chiot et al., 2023). Recently, a vectorized form of a full-length monoclonal antibody against TDP-43 (ACI-5891), delivered via adeno-associated virus 9, was shown to achieve sustained expression in mice for over 4 months. In a mouse model of ALS/FTD (CamKIIa-hTDP-43NLSm mice), treatment with the vectorized monoclonal antibody reduced levels of pathological phosphor-TDP-43 in neurons (Val et al., 2025). However, the functional impact of this reduction in protein aggregation remains to be elucidated.

The CD40 costimulatory pathway has been found to be upregulated in the peripheral blood of approximately 56% of ALS patients. The inhibition of CD40L with Tegoprubart (AT-1501) improves ALS clinical symptoms, including weight loss and paralysis, and extends survival in an ALS mouse model. Moreover, it delayed paralysis onset and extended survival in mSOD1^G93A^ mice (Lincecum et al., 2010; Ots et al., 2022). A phase 2a clinical trial assessed the safety and tolerability of Tegoprubart (AT-1501) across four ascending-dose cohorts (1, 2, 4, and 8 mg/kg) over 12 weeks. The study enrolled 54 ALS patients at 13 treatment centers in the United States and Canada. Tegoprubart (AT-1501) was well tolerated, with no safety concerns reported.

Several lymphocyte-based strategies have shown efficacy in preclinical models and clinical trials. However, selecting appropriate patient candidates remains a key challenge, requiring further characterization of disease-associated biomarkers.

### Immune cell therapy for Alzheimer’s disease

AD is a progressive neurodegenerative disorder characterized clinically by a gradual decline in cognitive function, most notably memory impairment, language difficulties, and executive dysfunction. The global prevalence of AD exceeds 50 million individuals, with projections indicating this figure may double every 20 years (Gustavsson et al., 2023). AD is characterized by extracellular Aβ deposition and intracellular hyperphosphorylated tau accumulation, forming senile plaques and neurofibrillary tangles, leading to cytotoxicity and neuronal death. In the early stages, patients may present with subtle memory lapses and difficulty performing complex tasks, which progressively worsen to include disorientation, confusion, personality changes, and impaired judgment. Diagnosis is primarily clinical, supported by cognitive assessments and neuroimaging techniques such as magnetic resonance imaging or positron emission tomography scans, and increasingly by biomarker analysis, including cerebrospinal fluid levels of amyloid-beta and tau proteins (Blennow and Zetterberg, 2018; Ferrari and Sorbi, 2021; Ossenkoppele et al., 2022). Despite the availability of symptomatic treatments, such as cholinesterase inhibitors and N-methyl-D-aspartate receptor antagonists, there is currently no cure for AD, highlighting the need for continued research into disease-modifying therapies.

Emerging evidence underscores the pivotal role of immune dysregulation in the pathogenesis and progression of AD, highlighting neuroinflammation as a core hallmark alongside Aβ and tau pathology (Heneka et al., 2015, 2025; Cai et al., 2025; Duggan et al., 2025). Both central and peripheral immune mechanisms contribute to this process: microglial activation, particularly through TREM2 signaling, and inflammasome pathways such as NLRP3, drive inflammatory responses that exacerbate neuronal dysfunction (Heneka et al., 2013; Ulland et al., 2017; Rachmian et al., 2024). Systemically, chronic low-grade inflammation or “inflammaging” characterized by elevated cytokines such as IL-6 and IL-1β has been associated with increased AD risk and progression. Longitudinal analyses have identified increased IL-1β and IL-6 as biomarkers in clinical AD stages, supporting their utility as predictive and therapeutic targets (Licastro et al., 2000). These insights have catalyzed the development of immunotherapies aimed at modulating specific immune pathways. Targeted approaches, including TREM2 agonists (e.g., AL002), CSF1R inhibitors (e.g., edicotinib), and TLR9 agonists (e.g., CpG1018), are under investigation for their potential to enhance microglial phagocytosis, suppress maladaptive activation, and promote neuroprotection (Heneka et al., 2015; Cai et al., 2022; Duggan et al., 2025). Nevertheless, distinct cell therapy approaches targeting the reduction of inflammatory phenotypes associated with AD are limited.

#### Macrophage-based therapy in Alzheimer’s disease

Microglia and peripheral monocytes/macrophages regulate the processes of neuroinflammation and Aβ clearance. Both respond to Aβ by becoming reactive cells, initially removing Aβ aggregates but eventually losing phagocytic activity and exhibiting proinflammatory signals, which are associated with the progression of AD (Gu et al., 2016; Munawara et al., 2021; Miao et al., 2023). Moreover, circulating peripheral monocytes/macrophages from healthy individuals without AD exhibits remarkably high expression of AD-associated genes, including *PTK2B*, *MEF2c*, *PFKFB2*, and others, suggesting a prodromal inflammatory profile predisposition (Raj et al., 2014). Thus, approaches focused on modulating microglial and monocyte polarization through epigenetic mechanisms from a pro-inflammatory (M1) to an anti-inflammatory (M2) phenotype have been proposed to be beneficial to modulate AD (Yao and Zu, 2020; Cisbani and Rivest, 2021).

In mice, it has been seen that transplantation of macrophages modified to overexpress angiotensin-converting enzyme, an Aβ-degrading peptidase, can reduce Aβ assemblies and improve cognitive performance in the AD-mice model (Koronyo-Hamaoui et al., 2020; **Figure 1**), suggesting that modified macrophages may mitigate deleterious phenotypes in AD. Furthermore, CAR cellular therapies show significant technological advancements. Injecting macrophages expressing a CAR targeting Aβ and producing M-CSF into the hippocampus can notably resorb Aβ and amyloid plaques in APP/PS1 transgenic mice (Kim et al., 2024; **Figure 1**). Additionally, targeting tau neurofibrillary tangles and various forms of Aβ plaques with different CAR antibodies presents promising therapeutic strategies against AD (Siebrand et al., 2025).

Although the macrophage-based approaches in AD remain limited, they show potential for therapeutic application. However, a deeper understanding of the underlying molecular pathways is required for successful clinical translation.

#### Lymphocytes-based therapy in Alzheimer’s disease

Evidence indicates that the peripheral adaptive immune system may have a role in AD. AD-transgenic mice treated with glatiramer acetate, a synthetic polypeptide that modulates the function of DCs to promote anti-inflammatory responses, exhibited CD11c^+^ DCs near amyloid plaques, containing intracellular Aβ. Contrarily, the elimination of bone-marrow-derived DCs resulted in increased amyloid plaque levels in these mice (Butovsky et al., 2007). Moreover, in transgenic AD mice, Aβ immunization led to the prominence of CD11c^+^ DCs and CD4 T cells in perivascular and leptomeningeal spaces of cerebral vessels with Aβ deposits (Fisher et al., 2011). This evidence suggests that peripheral immune cells attracted to Aβ deposits may play a role in their elimination, potentially reducing amyloid-β plaque formation and mitigating cognitive impairment in AD.

Moreover, observations in the 5XFAD mouse model have shown that reduced levels of interferon gamma (IFN-γ) and chemokines ICAM-1, VCAM-1, CXCL10, and CCL2 in the choroid plexus correlate with decreased leukocyte trafficking to the central nervous system. This phenomenon is partly attributed to the heightened activity of immunosuppressive Foxp3^+^ Tregs. Consequently, transient depletion of Treg cells facilitates the recruitment of immunoregulatory cells to Aβ plaques, promoting their clearance, mitigating neuroinflammation, and rescuing cognitive decline (Baruch et al., 2015; Yeapuri et al., 2023). Comparable outcomes were observed following the inhibition of the programmed death-1 pathway, which elicited an IFN-γ-dependent systemic immune response and recruited monocyte-derived macrophages to the brain (Baruch et al., 2016).

Although lymphocytes and DCs play active roles in the AD progression, direct therapies targeting these cells are uncommon. However, some evidence suggests potential in lymphocyte-based therapies, such as using peripheral macrophages and natural killer cells to enhance Aβ clearance and reduce neuroinflammation (Xu and Jia, 2021; Kostic et al., 2025). Furthermore, CAR T cells engineered to target Aβ aggregates represent a novel and targeted approach under preclinical investigation (Rajesh and Kanneganti, 2022; Pfeffer et al., 2025; **Figure 1**). Additionally, a similar strategy using engineered T cell receptors specific for Aβ in Treg cells (TCR Aβ-Tregs) has been developed. Transferring these TCR Aβ-Tregs to APP/PS1 AD-mouse led to lasting immune suppression, reduced microglial activity, and lower amyloid loads (Yeapuri et al., 2023; **Figure 1**). These innovative strategies highlight the therapeutic potential of harnessing both arms of the immune system to combat AD and pave the way for the development of disease-modifying treatments.

One of the most advances in AD immunotherapy is the development of monoclonal antibodies targeting Aβ or phosphorylated tau as a passive immunization (**Figure 1**). Aducanumab (BIIB037; Aduhelm™) is an IgG1 monoclonal antibody that binds the N-terminal epitope (aminoacids 3–7) of the Aβ_42_ peptide. In transgenic AD mouse models and human patients, aducanumab penetrates the brain, bind parenchymal Aβ, and reduce both soluble and insoluble Aβ in a dose-dependent manner (Sevigny et al., 2016). Approved by the U.S. Food and Drug Administration, aducanumab became the first monoclonal antibody targeting Aβ and the first disease-modifying therapy approved for AD. It is recommended for early-stage AD patients with mild cognitive impairment or mild dementia who exhibit Aβ pathology confirmed by amyloid positron emission tomography or CSF analysis. Donanemab (Kisunla™) is another anti-amyloid antibody used for early AD in patients with mild cognitive impairment of dementia and high brain Aβ levels (Cummings et al., 2024). Similarly, Lecanemab (Leqembi®) targets amyloid-beta plaques and has demonstrated efficacy in reducing plaque burden and slowing cognitive decline in individuals with mild cognitive impairment or early AD (Cummings et al., 2024). Finally, gantenerumab targets Aβ fibrils by recognizing amino acids 3–11 and 18–27 of Aβ. Phase 1, 2, and 3 trials have evaluated its safety, efficacy, and pharmacodynamics, showing that while it reduces amyloid plaques, it does not significantly impact cognitive decline (Bateman et al., 2022; Kim et al., 2025).

Although the administration of antibodies demonstrates promise as a strategy for modifying AD, single-chain variable fragments (scFv) derived from these or other antibodies can be utilized to alter immune cells, thereby enhancing their anti-inflammatory properties and their ability to clear amyloid. Recent proof-of-concept studies have developed CAR-targeting Aβ in Treg cells, which exhibited typical Treg characteristics, could be activated, and demonstrated suppressive abilities (Saetzler et al., 2023). Moreover, this strategy has demonstrated selectivity toward various aggregates (such as Aβ_1-42_, Aβ_p3-42_, and Tau neurofibrillary tangles) contingent upon the specific AD antibody-based CAR employed (Siebrand et al., 2025). However, its efficacy as a disease-modifying therapy for AD has yet to be established.

Taken together, several approaches have been explored in the search for disease-modifying cell-based immunotherapy for AD. A key challenge remains validating these findings in clinical trials.

### Immune cell therapy for Parkinson’s disease

PD is the second most common neurodegenerative disease with an approximated prevalence of 3% in 60-year-old population (Lee and Gilbert, 2016; Balestrino and Schapira, 2020; Simon et al., 2020). It is characterized by motor symptoms (bradykinesia, resting tremor, muscle rigidity) and non-motor symptoms (such as sleep disturbance and gastro-intestinal dysfunction) and its etiology is considered multifactorial, where age is a major risk factor for developing PD (Simon et al., 2020). Only a few cases (5%–10%), referred to as familial PD, are associated with mutation or genetic variants. In contrast, the vast majority have unknown origins and are classified as sporadic PD (Lee and Gilbert, 2016; Simon et al., 2020). PD is characterized by the progressive degeneration of dopaminergic (DA) neurons at the *Substantia Nigra pars compacta*, accompanied by the formation of Lewy bodies, which are protein inclusions composed primarily of misfolded α-syn (Spillantini et al., 1997; Farrer, 2006). This aberrant intracellular accumulation is largely attributed to disrupted protein homeostasis, leading to selective DA neuron loss and a decline in brain dopamine levels, which manifest as PD-related symptoms (Erkkinen et al., 2018; Simon et al., 2020). By the time clinical symptoms emerge, approximately 50%–70% of *Substancia Nigra pars compacta* DA neurons have already degenerated (Fearnley and Lees, 1991), rendering current treatment unable to halt or delay disease progression (Kakkar and Dahiya, 2015; Troncoso-Escudero et al., 2020). To address this challenge, extensive research has focused on elucidating the molecular mechanism driving DA neuron vulnerability. Key factors include impairment in protein degradation pathways, neurotrophic factor deficiencies, mitochondrial dysfunction and oxidative stress, as well as exacerbated neuroinflammatory responses. All of which interact in a complex pathological network (Phani et al., 2012; Troncoso-Escudero et al., 2020). Currently, inflammation emerges as a key contributor involved in PD progression, supported by patient studies showing enhanced pro-inflammatory cytokines in brain tissue (Kouli et al., 2020) and cerebrospinal fluid (Mogi et al., 1994, 1996; Blum-Degen et al., 1995; Hirsch et al., 2012), indicative of heightened neuroinflammation. Moreover, elevated levels of proinflammatory cytokines have also been found in peripheral blood mononuclear cells (Munoz-Delgado et al., 2023; Grunenwald et al., 2025) and serum (Scalzo et al., 2010), with a positive correlation to disease severity and progression (Ahmadi Rastegar et al., 2019). Beyond cytokine presence, the activation of glial cells (microglia and astrocytes), and their involvement in PD pathogenesis as well as the contributions of peripheral immune cells to the pro-inflammatory environment in the brain, have been widely reported (Gerhard et al., 2006; Hirsch et al., 2012; George et al., 2019; Grunenwald et al., 2025), further implicating immune dysregulation in disease development.

#### Macrophage-based therapy in Parkinson’s disease

Monocytes/macrophages have been implicated in PD pathology. During neurodegeneration, peripheral monocytes/macrophages infiltrate the CNS in animal models of PD-like degeneration (Tentillier et al., 2016; Harms et al., 2017, 2018) and in the brains of PD patients (Pey et al., 2014). Moreover, monocytes/macrophages exhibit altered secretion patterns of proinflammatory cytokines, including IL-1α, IL-1β, TNF-α, IL6, and IFN-γ, correlating with PD severity (Hasegawa et al., 2000; Reale et al., 2009). Additionally, evidence suggests impaired function, as these cells display or proinflammatory pre-disposition to inflammatory stimuli (Grozdanov et al., 2014). Studies of peripheral monocytes in PD patients have shown enrichment of classical monocytes expressing CCL2 in blood, with elevated CCL2 levels in the CSF (Funk et al., 2013; Grozdanov et al., 2014). Furthermore, circulating monocytes from healthy individuals without PD exhibit remarkably high expression of PD-associated genes, including *α-syn*, *LRRK2*, and others (Raj et al., 2014).

Peripheral monocytes/macrophages have been explored as a cell-based therapy in PD, primarily due to their migratory capacity and role as cellular carriers (**Figure 1**). Building on this concept, Biju J. and their colleagues developed a method using modified bone marrow stem cell-derived macrophages engineered to deliver glial cell line–derived neurotrophic factor (GDNF), a potent neuroprotective agent, which has challenges in crossing the BBB. In this approach, bone marrow stem cells were transduced *ex vivo* with lentivirus expressing a GDNF gene driven by a macrophage-specific promoter, then transplanted into recipient mice (1–2 × 10^6^ cells per intravenous injection). Eight weeks post-transplantation, mice were injected with neurotoxin (1-methyl-4-phenyl-1,2,3,6-tetrahydropyridine; MPTP) for 7 days to induce dopaminergic neurodegeneration. Macrophage-mediated GDNF therapy mitigated MPTP-induced degeneration of tyrosine hydroxylase (TH)-positive neurons of the *Substantia Nigra* and TH^+^ terminals in the striatum, reversing hypoactivity in the open field test (Biju et al., 2010). Following a similar method, hematopoietic stem cell transplantation combined with macrophage-mediated delivery of GDNF was administered (3 × 10^6^ cells per intravenous injection) after body irradiation to treat MitoPark mice, a PD preclinical model. After 9 weeks, this treatment significantly improved motor and non-motor symptoms, reduced dopaminergic neuron loss, and preserved striatal axonal terminals (Chen et al., 2018). Additionally, syngeneic bone marrow HSCs were transduced *ex vivo* with a lentiviral vector to express GDNF and transplanted (2 × 10^6^ cells per intravenous injection) into 14-week-old MitoPark mice. This resulted in increased GDNF levels in the midbrain via infiltration of transplanted cells, mitigating dopaminergic neuron loss in both the *Substantia Nigra* and the ventral tegmental area while preserving axonal terminals and dopamine levels in the striatum (Chen et al., 2020).

Peripheral macrophages have also been used as carriers for delivering therapeutic molecules to distinct brain regions (**Figure 1**). Bone-marrow-derived macrophage load with nanozyme particles of active catalase were transplanted (10 × 10^6^ cells per intravenous injection) in an MPTP model. These cells were detected in the brain, spleen, liver, lung, kidney within 24 hours, contributing to a reduction in oxidative stress in this model (Batrakova et al., 2007). A subsequent detailed biodistribution analysis of intravenously injected bone-marrow-derived macrophage loaded with nanozyme-catalase (5 × 10^6^ cells per intravenous injection) revealed that these cells are present in blood up to 120–168 hours and reached the spleen, liver, and brain within 1 hour, mitigating microgliosis and preserving TH^+^ neurons in this PD model (Brynskikh et al., 2010).

Given that macrophages can infiltrate brain tissue and express CD163 marker, dexamethasone-loaded liposomes targeted for the CD163 receptor were carried into the brain after peripheral intravenous injection in 6-hydroxydopamine-lesioned rats, a PD preclinical model. This treatment improved motor performance with no observed side effects, and partially enhanced TH^+^ neuron survival, correlating with a distinctive microglia response (Tentillier et al., 2016).

An innovative approach involves the use of extracellular vehicles (EVs) derived from autologous macrophages (**Figure 1**). Exosomes from inflamed macrophages stimulate proinflammatory cytokine expression in microglia and astrocytes, leading to neuroinflammation. In animal models, EVs from inflamed macrophages treatment induce PD-like symptoms, including motor deficits and loss of dopaminergic neurons in the *Substantia Nigra* and striatum. Exosomal miRNAs, particularly miR-155-5p, have been identified as key mediators of this inflammatory response (Jin et al., 2023). Expanding on this concept, EVs from modified GDNF-macrophages (EV-GDNF), carrying both GDNF protein and its encoding DNA, have been investigated. In a Parkin Q311X(A) mouse, a PD preclinical model, intranasal administration of EV-GDNF (once a week during 3 weeks, 3 × 10^9^ particles) significantly improved motor function, increased neuronal survival, and reduced neuroinflammation, including decreased microgliosis and proinflammatory cytokine levels, with no observed toxicity (Zhao et al., 2022).

Other interesting growth factor to PD treatment is insulin-like growth factor 2 (IGF2), which modulates the innate immune response of macrophages, establishes an oxidative phosphorylation metabolism, and an anti-inflammatory phenotype (Du et al., 2019; Wang et al., 2020). IGF2 is a secreted factor with neuroprotective properties in several models of neurodegenerative disease (Mellott et al., 2014; Garcia-Huerta et al., 2020; Alberini, 2023; Arcos et al., 2023; Fitzgerald et al., 2023; Tung et al., 2025). Additionally, we reported a significant decrease in IGF2 levels in both plasma and peripheral blood mononuclear cells in PD patients (Sepulveda et al., 2022). Recently, our group reported the potential therapeutic use of IGF2-reprogrammed macrophages (MIGF2) in PD. IGF2 treatment prevented the proinflammatory phenotype induced by α-syn preformed fibril exposure in murine primary macrophages by reducing the inflammatory response. Notably, intravenous treatment with MIGF2 (2 × 10^5^ cells, once a week for 4 weeks) significantly reduced motor impairment, decreased α-syn accumulation, and diminished microglial activation in the *Substantia Nigra* of a preclinical PD model at early and late stages of disease progression (Grunenwald et al., 2025; **Figure 1**).

Several macrophage-based therapies have been explored for PD, all demonstrating significant therapeutic potential. However, further validation of these preclinical findings in clinical trials is necessary.

#### Lymphocytes-based therapy in Parkinson’s disease

The adaptive immune response, consisting of T and B lymphocytes, generates long-lasting protection against specific foreign antigens. Following activation of T cells generates a specific subtype of CD4 T helper (CD4 Th) cells or CD8 cytotoxic (Mosley et al., 2012; Baird et al., 2019). An increase of CD4 and CD8 T cells in the *Substancia Nigra pars compacta* of PD patient post-mortem brain, localized around DA neurons or near blood vessels, reveals that T cell infiltration plays a role in the neuroinflammation process described in PD (Brochard et al., 2009). Also, peripheral B cell activation leads to antibody secretion that crosses the blood-brain barrier and is deposited in the CNS in response to plasmatic α-syn in a PD preclinical model (Theodore et al., 2008). Additionally, Tregs, which are capable of decreasing neuroinflammation and DA neuron loss, are functionally arrested in PD patients (Gruden et al., 2011; Stevens et al., 2012; Li et al., 2022). The physiological role of Tregs is to suppress specific immune responses by releasing anti-inflammatory cytokines such as IL-10 and transforming growth factor β, inhibiting antigen presentation, attenuating Th2 and Th17 function, and dampening microglia activation (Reynolds et al., 2007; Stone et al., 2009). Accordingly, Treg dysfunction has been linked with decreased motor coordination and increased PD severity (Saunders et al., 2012).

The current immunoregulatory therapies designed for the treatment of PD follow two different strategies. The first approach, active immunization, consists of stimulating the immune system to produce antigen-specific antibodies to support the removal of α-syn aggregates from blood and CNS through the generation of an immune response towards the immunizing agent involving T and B cells (Masliah et al., 2005; Bae et al., 2012; von Euler Chelpin and Vorup-Jensen, 2017). A second approach involves modifying peripheral immune cells (**Figure 1**). For example, granulocyte macrophage-colony stimulating factor (GM-CSF) treatment results in increased frequencies of Treg and Treg function in PD patients, suggesting that immune modulation with GM-CSF could mediate immune responses for potential therapeutic treatment (Gendelman et al., 2017). Also, adoptive transfer of dendritic cells treated with GM-CSF increased splenic Tregs, attenuated neuroinflammatory responses, and protected nigrostriatal DA neurons in a PD preclinical model (Schutt et al., 2018). Additionally, adoptive transfer of Treg cells has been explored in experimental models of PD. Adoptive transfer of T cells (5 × 10^7^ splenocytes; intravenous injection) from Copolymer-1-immunized mice administered to MPTP recipients could slow disease progression, improving TH^+^ neuronal survival by suppression of microglial activation, and increased local expression of astrocyte-associated GDNF (Benner et al., 2004). Moreover, following the same concept, adoptive transfer of CD4^+^ T cells or CD3^+^CD4^+^CD25^+^ Tregs (3.5–5 × 10^6^ cells, intravenously injection) exhibited a protection of the nigro-striatal system in a MPTP mouse model, primarily by also modulating microglial responses, decreasing α-syn aggregation, and up-regulating GDNF and transforming growth factor β (Laurie et al., 2007; Reynolds et al., 2007). Furthermore, combinations of adoptively transferred vasoactive intestinal peptide immunocytes (5 × 10^7^ cells; intravenously injection) or natural Treg (1 × 10^6^ Treg cells; intravenously injection) administered to MPTP mice immunized with nitrated α-syn, attenuated Th17 immune responses, a microglial inflammatory responses and led to robust nigrostriatal protection (Reynolds et al., 2010; **Figure 1**). Moreover, the development of antibodies to target and neutralize CD95L (CD95-Fc fusion protein), a protein expressed in peripheral monocytes, has been shown to improve TH^+^ neuronal survival and decrease microgliosis in a preclinical model of PD (Gao et al., 2015).

In addition, using a lipid nanoparticle delivery system containing Gm-csf mRNA to boost the number and function of Tregs (**Figure 1**). Restoring Treg activity is known to suppress neuroinflammation and protect neurons. In preclinical PD models, administration of *Gm-csf* mRNA led to dose-dependent increases in GM-CSF levels, enhanced CD4^+^CD25^+^FoxP3^+^ Treg populations, and nigrostriatal neuroprotection. The treatment also reduced microglial activation and induced immunosuppressive gene expression (Olson et al., 2021).

The use and modulation of lymphocytes have demonstrated therapeutic potential for PD. However, translating these findings into clinical applications will be crucial for developing effective strategies.

### Immune cell therapy for Huntington’s disease

HD is a progressive and irreversible neurodegenerative disorder inherited in an autosomal-dominant manner, caused by a trinucleotide (CAG)_n_ repeat expansion in the coding sequence of the *IT-15* gene, which encodes the huntingtin protein (Htt) (Jimenez-Sanchez et al., 2017; Jiang et al., 2023). In recent years, the incidence has been reported as 0.48 cases per 100,000 person‐years, with a higher prevalence in Europe and North America (Medina et al., 2022). The Htt mutation (mHtt) results in an abnormal polyglutamine (> 37Q, polyQ) repeat length (van der Burg et al., 2009; Jiang et al., 2023). Expanded polyglutamine (polyQ) leads to misfolding and aggregation of Htt in neurons, primarily in medium spiny neurons of the dorsal striatum. This disrupts cellular functions through mitochondrial dysfunction, oxidative stress, inflammation, and synaptic dysfunction (van der Burg et al., 2009), ultimately affecting motor control and cognitive function (Illarioshkin et al., 2018). While strategies targeting mHtt aggregation show some promise, they have not yet prevented disease progression, indicating the need for broader therapeutic approaches (Davies and Turmaine, 1997; Yamamoto et al., 2000; Moore and Armstrong, 2003).

Recent evidence indicates that chronic immune activation is a pathological hallmark of HD, reflecting a complex interplay between misfolded protein aggregates and immune response (Ellrichmann et al., 2013; Rocha et al., 2016; Fatoba et al., 2020). mHtt is recognized by both resident and peripheral immune cells, where it serves as a pro-inflammatory stimulus (Crotti et al., 2014; Fatoba et al., 2020). Moreover, mHtt is endogenously expressed and detectable within these immune cell populations (Weiss et al., 2012; Crotti et al., 2014). Increase microglial activation and elevated proinflammatory cytokines (TNF-α, IL-6, and IL-8) in plasma, CSF, and brain tissue, along with chemokine dysregulation, have been widely reported in HD patients and rodent models, correlating with disease progression (Sapp et al., 2001; Rocha et al., 2016). Additionally, mHtt interacts with cellular pathways to induce proteosome dysfunction and inflammation, as observed in mouse neuroblastoma cells expressing mHtt, which exhibit significantly elevated proinflammatory-associated proteins, including monocyte chemoattractant protein-1 (Godavarthi et al., 2009). Moreover, mHtt influences NF-κB signaling, a key regulator of inflammatory responses. Activation of IκB kinases releases NF-κB from cytoplasmic sequestration, leading to nuclear translocation and upregulation of proinflammatory-associated genes. mHtt has been shown to interact with IkB and upregulate the IκB kinases and NF-κB activation in immune cells from human and preclinical HD model (Hsiao et al., 2013; Trager et al., 2014; Khoshnan et al., 2017). Collectively, these findings strongly support the involvement of inflammation in HD pathology and its contribution to disease progression.

#### Macrophage-based therapy in Huntington’s disease

As mentioned earlier, HD involves profound immune dysfunction, including in monocytic lineage. Peripheral monocytes/macrophages and microglia from human HD patients and preclinical models exhibit dysfunctional inflammatory phenotypes, characterized by a primed pro-inflammatory profile with excessive IL-6 and TNF-α expression, heightened responses to inflammatory stimuli (lipopolysaccharide and IFN-γ), and impaired migration and phagocytosis. Interestingly, mHtt levels in peripheral monocytes/macrophages have been significantly associated with disease progression markers, including whole-brain atrophy and ventricular expansion (Weiss et al., 2012). Furthermore, this dysfunctional inflammatory profile may be counteracted by reducing endogenous Htt/mHtt expression via siRNA (Trager et al., 2014; O’Regan et al., 2020; **Figure 1**), suggesting that Htt plays a crucial role in macrophage function regardless of their activation state, potentially regulating key inflammatory signaling pathways such as NF-κB and associated gene expression. However, further *in vivo* investigation is needed to fully elucidate the impact of Htt on the inflammatory profile.

These findings highlight new therapeutic possibilities centered on targeting monocytes/macrophages, expanding the scope of cellular-based treatments for HD.

#### Lymphocytes-based therapy in Huntington’s disease

The involvement of the lymphocytic lineage in HD pathology remains a significant challenge in establishing new therapeutic targets (Fatoba et al., 2020). Key inflammatory mediators of T and B cells, such as IL-4, are altered in the serum of HD patients, suggesting a dysregulation within these lineages (Bjorkqvist et al., 2008). Additionally, similar to monocytes, T and B cells from HD patients also express mHtt, with levels increasing as the disease progresses and correlating with disease burden scores (Weiss et al., 2012), a measure that integrates the individual’s age and Htt CAG repeat to estimate clinical progression (Penney et al., 1997; Tabrizi et al., 2012). However, this evidence contrasts with reports indicating that T and B cell populations (Miller et al., 2015a; Trager et al., 2015), plasma immunoglobulin levels, proliferation, cytokine secretion, and transcriptional inflammatory profiles of T cells (Bjorkqvist et al., 2008; Miller et al., 2015a) remain unchanged. Despite these discrepancies, HD patients may exhibit distinct dysfunctions within these cells, warranting further investigation.

Among the most studied approaches involving immunotherapy in HD is the use of antibodies targeting mHtt (**Figure 1**), which is present both intracellularly within neurons and extracellularly in the CSF, plasma, and extracellular matrix (Jamwal et al., 2020). Passive immunization, involving the administration of antibodies or intrabodies, has been employed to mHtt. Intrabodies are small single- or double-antibody fragments (140–250 amino acids in length) that specifically bind intracellular antigens with high affinity for defined epitopes. These molecules can be engineered and delivered either as genes or as recombinant proteins (Messer and Joshi, 2013). The therapeutic potential of this technology targeting mHtt on HD has been widely addressed in cell culture and HD preclinical rodent models. Intrastriatal delivery of single-chain Fv-C4 intrabody (scFv-C4), targeting the first 17 amino acids of the N-terminal fragment of mHtt (Lecerf et al., 2001; Murphy and Messer, 2004; Miller et al., 2005), via adeno-associated virus 2/1 vector, delayed mHtt accumulation in B6.Cg-HDR6/1 transgenic mice (Snyder-Keller et al., 2010) and in an HD Drosophila model (Wolfgang et al., 2005). Additionally, fusing scFv-C4 with a PEST motif region of mouse ornithine decarboxylase enhances its efficacy in reducing mHtt aggregation and toxicity (Butler and Messer, 2011). Other intrabodies targeting distinct mHtt domains have also been identified, including VL12.3 (targeting the N-terminal domain) (Southwell et al., 2008), mEM48 (targeting Valine-Alanine residues in exon 1) (Wang et al., 2008), Harpp1 (targeting proline-rich domain) (Southwell et al., 2009), and INT41 (targeting proline-rich on the carboxyl (3’) side of the polyglutamine expansion) (Amaro and Henderson, 2016). Furthermore, recognizing mHtt prion-like properties shifts the paradigm of antibody-based therapies, as targeting extracellular mHtt could represent a disease-modifying strategy (Jansen et al., 2017). Considering this concept, C6-17 monoclonal antibody, which targets an exposed region of Htt near the aa586 Caspase 6 cleavage site that effectively blocks cell-to-cell propagation of mHtt *in vitro* has been developed (Bartl et al., 2020), which reduced mHtt levels while delaying HD symptoms, including weight and motor impairments (Bartl et al., 2024; **Figure 1**).

Conversely, active immunotherapy has been less explored in HD, which states for the synthesis and secretion of endogenous antibodies targeting mHtt. A previous study has shown that intramuscular DNA vaccination using 5’ fragment of the mHtt cDNA ameliorate glucose intolerance and the diabetic phenotype in R6/2 mice, a well-established preclinical model of PD (Miller et al., 2003). Furthermore, immunization with a combination of three non-overlapping Htt exon 1 peptides (AA1-17, AA49-60, AA74-88; conjugated to KLH with alum adjuvant), elicited a strong immunogenic response against Htt-targetted epitopes in two different HD preclinical models (Ramsingh et al., 2015; **Figure 1**). Remarkably, immunized mutant HD mice present a differential expression of immune-related genes, highlighting an up-regulation of innate immune responses and down-regulation of helper and memory T cell responses, including regulation of NF-κB activation and cytokine-chemokine signaling (Ramsingh et al., 2015).

These findings suggest that both passive and active immunotherapies targeting are promising for treating HD, nevertheless, further preclinical studies to assess immunogenicity, safety, and efficacy in HD models are needed.

## Concluding Remarks

Various approaches have been explored to treat neurodegenerative disease, particularly through cell-based immunotherapy as a potential disease-modifying strategy. Despite extensive efforts, no therapy has fully halted disease progression, though some have shown promise in preclinical and phase I–III clinical trials. The complexity of patient populations-considering genetic predisposition and environmental factors adds further challenges to effective implementation. Additionally, differences between preclinical models and patients associated with aging, genetic, and environmental factors contribute to clinical trial failures, where immunotherapy-based treatments often show minimal or no disease-modifying effects.

Future strategies may benefit from combining multiple immunotherapy targets. Such as immunomodulators alongside *ex vivo*-modified immune cells such as T cells or macrophages, to enhance therapeutic efficacy. Given the complexity of the human brain and neurodegenerative phenotypes, current classifications may be insufficient for selecting appropriate patients for tailored treatment. Developing a more comprehensive biomarker panel could be essential for refining immunotherapy approaches and targeting specific patient immunophenotypes.

Furthermore, targeting solely the immune system, whether innate or adaptive, may be insufficient, as neurodegenerative disease involves both inflammatory processes and cell-autonomous pathological mechanism affecting specific neuronal populations and brain regions. An integrated, multiscale therapeutic model tailored to individual disease phenotypes may be the most effective approach. Combining cell-based immunotherapy with therapies targeting neurons could lead to a synergistic effect, potentially halting or altering disease progression.

As this field evolves, cell-based immunotherapy is emerging as a cornerstone of personalized medicine, offering hope for patients with refractory, progressive, or irreversible diseases and paving the way for the next-generation immunotherapies in neurodegenerative disorders, as seen in other pathologies.

## Data Availability

*Not applicable.*
